# Pathological Affective Dependence as a Risk Factor for Intimate Partner Violence: Initial Psychometric Validation of the Italian Version of the Pathological Affective Dependence Scale

**DOI:** 10.1002/cpp.70140

**Published:** 2025-08-15

**Authors:** Pugliese Erica, Uvelli Allison, van Emmerik Arnold, Ferretti Fabio, Saliani Angelo Maria, Foschino‐Barbaro Maria Grazia, Vigilante Teresa, Celitti Erika, Quintavalle Chiara, Mancini Francesco, Arntz Arnoud

**Affiliations:** ^1^ Clinical Psychology Group, Faculty of Social and Behavioural Sciences University of Amsterdam Amsterdam the Netherlands; ^2^ School of Cognitive Psychology Rome Italy; ^3^ Department of Medical Science, Surgery and Neurosciences University of Siena Siena Italy; ^4^ School of Cognitive Psychology (APC‐SPC) Rome Italy; ^5^ School of Cognitive Psychology (AIPC) Bari Italy; ^6^ University Guglielmo Marconi Rome Italy

**Keywords:** assessment, intimate partner violence, pathological affective dependence, survivors, validation

## Abstract

**Purpose:**

This study aimed to validate the Italian version of the Pathological Affective Dependence Scale (PADS), which assesses both trait and state manifestations of Pathological Affective Dependence (PAD), a key risk factor for Intimate Partner Violence (IPV).

**Methods:**

Two studies were conducted in the general population and IPV survivor samples. Study 1 used a sample of 360 participants for Confirmatory and Exploratory Factor Analysis (CFA and EFA). Study 2 included 362 participants for an additional CFA and reliability analysis to further assess the scale's factor structure and internal consistency, and for a correlational analysis to determine its convergent and discriminant validity.

**Results:**

Our factor analytic findings revealed that the PADS' 17 items represent three factors: internal conflict, inability to separate and partner abuse. The scale's state and trait versions showed satisfactory reliability, and convergent and discriminant validity.

**Conclusions:**

The findings suggest that the PADS is a useful tool for assessing and enhancing our understanding of PAD as a psychological risk factor for IPV. As such, it can help prevent IPV and support individual IPV survivors by identifying and treating PAD, while also contributing to clinical and social research into IPV.

## Summary

- Pathological Affective Dependence (PAD) may help explain why individuals remain in abusive relationships.
- The PADS is a psychometric tool developed to assess PAD in clinical and research settings.
- The PADS showed good reliability and construct validity in Italian samples.
- Assessing PAD can support clinicians in identifying psychological risk factors underlying Intimate Partner Violence and guiding targeted interventions.

## 1 Introduction

The World Health Organization (WHO 2024) defines Intimate Partner Violence (IPV) as a ‘behavior by an intimate partner or ex‐partner that causes physical, sexual, or psychological harm, including physical aggression, sexual coercion, psychological abuse, and controlling behavior’. These forms of violence have well‐documented consequences for both physical and mental health (Whiting et al. 2016; Karakurt et al. 2016; Zancan and Habigzang 2018; Bacchus et al. 2018; Potter et al. 2020; Oram et al. 2022; Wessells and Kostelny 2022; Uvelli et al. 2024) and negatively affect survivors' children, who may witness or otherwise become aware of the violence (Foschino Barbaro et al. 2023; Oliver and Jaffe 2023).

## 2 Conceptualization of Pathological Affective Dependence (PAD)

Among the psychological factors that may contribute to the persistence of IPV, PAD is increasingly recognized as a critical mechanism for understanding why some individuals remain in abusive relationships (Crapolicchio et al. 2021; Pugliese, Saliani, et al. 2023). In 2023, Pugliese and colleagues introduced a first cognitive model of PAD, defining it as an excessive attachment of a person to an abusive, violent or manipulative partner that is characterized by an overwhelming belief that maintaining the relationship with this partner is essential (Pugliese, Saliani, et al. 2023; Pugliese, Mosca, et al. 2023). The defining characteristic of PAD is the presence of an internal conflict between the goal of separating to ensure one's safety versus the goal of maintaining or saving the relationship at all costs. The insight a person has in this conflict can be categorized as absent, alternating or akratic. Absent conflict refers to situations in which others recognize the dysfunctional nature of a harmful relationship, while the person remains unaware of this and perceives the advantages of the relationship as outweighing the disadvantages. Alternating conflict is marked by fluctuations between opposing intentions to leave or remain in a harmful relationship, preventing a definitive decision. Akratic conflict arises when a person acknowledges the need to leave a harmful relationship but feels emotionally incapable of doing so, leading to paralysis and sustained dependence. PAD may characterize a current or recent relationship (state condition) or constitute a pattern across successive relationships (trait condition) (Pugliese, Mosca, et al. 2023).

The development of insight in survivors of violent relationships often follows a sequential pattern: initial unawareness of the abuse, followed by a phase of ambivalence marked by alternating recognition and denial and finally, full awareness of the violence and the need to leave. However, in the presence of PAD, this awareness may coexist with a strong emotional dependence, generating an internal conflict between self‐protection and the urge to preserve the bond.

The cognitive model of PAD (Pugliese, Saliani, et al. 2023; Pugliese 2024) postulates that the early frustration of three basic needs of love, dignity and safety lies at the root of the development of PAD. Examples include being forced to cope with depressed and unresponsive parents (lack of love), invalidating or devaluing parents (lack of dignity) and emotional or physical neglect or parental IPV (lack of safety). The model distinguishes between distal and proximal factors that frustrate these basic needs. Distal factors refer to early relational experiences that leave the need for love, security and dignity unmet, particularly in childhood or formative relationships, and that contribute to the development of maladaptive attachment patterns (PAD trait). In contrast, proximal factors refer to more recent or current frustrations of basic needs due to the ongoing partner abuse (PAD state). These distal and proximal factors may contribute to and perpetuate PAD, making it challenging for a person to break free from a harmful relationship.

It is important to note that PAD is only one of multiple barriers to leaving such relationships; perhaps the most immediate and powerful obstacle is the pervasive fear induced by the violent partner's threats and intimidation, which profoundly complicates survivors' ability to escape abusive situations. Indeed, leaving an abusive partner is a high‐risk moment, often associated with severe injury, femicide and risks to children (McFarlane et al. 2004; Bell et al. 2007).

## 3 Distinguishing PAD From Related Constructs

Although PAD shares certain surface‐level similarities with Dependent Personality Disorder (DPD) and behavioural addictions—such as compulsive attachment and difficulty terminating relationships (Kwee 2007; Reynaud et al. 2010)—its underlying motivational and relational dynamics are notably distinct. Unlike individuals with DPD, who typically seek emotionally stable and responsive partners to meet generalized needs for reassurance and support, individuals with PAD are often unconsciously drawn to partners who mirror dysfunctional characteristics of early attachment figures. This pattern is rooted in unresolved relational trauma, where love is internalized as being conditional, inconsistent or intertwined with control and neglect (Fonagy et al. 2018). Such dynamics exemplify a repetition compulsion (Levy 2000), whereby the individual unconsciously attempts to ‘repair’ earlier emotional wounds by recreating and mastering familiar, yet harmful, relational scenarios. In this context, the abusive or emotionally unavailable partner is paradoxically perceived as the key figure who might finally fulfill early unmet needs for love, validation or safety—thus reinforcing the dependent attachment.

Although PAD can manifest with features common to behavioural addictions—such as emotional craving, withdrawal symptoms and compulsive relational pursuit—its motivational architecture is more complex. The drive is not merely to avoid emotional pain or seek short‐term gratification, but rather to fulfill an unconscious hope for emotional repair. The abusive partner is seen not just as an object of desire, but as a symbolic redeemer of unmet developmental needs, making the attachment uniquely difficult to relinquish (Urbiola et al. 2017; Valle and Moral Jiménez 2018). A further distinction lies in the fact that PAD may present either as a state condition, tied to a specific abusive or manipulative relationship, or as a trait condition, reflecting a recurring pattern of maladaptive relational dependence across the lifespan (Pugliese, Mosca, et al. 2023). This dual expression contrasts with most personality disorders and behavioural addictions, which tend to follow a more stable trait‐like course, highlighing the need for clinical tools that can assess both situational vulnerability and enduring personality dynamics in affective dependence.

Taken together, PAD can be conceptualized as a trauma‐related relational schema characterized by a profound internal conflict between the instinct for self‐protection and the compulsive drive to preserve a harmful attachment. These dynamics distinguish PAD from both DPD and addiction models, underscoring its specificity and relevance for understanding the psychological mechanisms that contribute to the maintenance of violent relationships.

## 4 Rationale for the Current Study

According to the WHO (2024), 27% of women aged 15–49 worldwide have experienced physical and/or sexual violence by a partner at least once in their lifetime. In Italy, recent data from ISTAT (2024) report that 113 women were killed in the past year, 99 of whom were murdered in a family or intimate context; in 61 of these cases, the perpetrator was a current or former partner. Notably, more than half (51.3%) of the survivors had experienced prolonged abuse, often resulting in severe mental health consequences such as anxiety, trauma‐related symptoms and psychological subjugation.

Despite the high prevalence of IPV, there remains a gap in clinically grounded tools capable of capturing the internal psychological dynamics—such as affective dependence—that may help explain why survivors struggle to disengage from abusive partners. Addressing this gap is crucial for accurate clinical assessment and developing targeted prevention strategies.

The Pathological Affective Dependence Scale (PADS) was developed to assess PAD both as a state condition—linked to a specific abusive relationship—and as a trait condition—reflecting a recurrent pattern of maladaptive relational dependence. The scale is grounded in the cognitive model of PAD proposed by Pugliese, Saliani, et al. (2023) and in the theory of goal‐directed behaviour (Miller 1960; Castelfranchi and Parisi 1980; Miceli and Castelfranchi 1995; Weiner 2010). This theoretical framework conceptualizes human behaviour as regulated by interconnected belief–desire–intention systems, in which affective and cognitive appraisal processes shape the persistence or disengagement from specific goals. Within this perspective, PAD reflects the rigid and maladaptive pursuit of relational goals perceived as essential for psychological survival—even when the relationship becomes a source of harm.

## 5 Aims of the Present Study

This study examines the internal structure, internal consistency and convergent and discriminant validity of the PADS in the Italian context. To ensure broad applicability and robust psychometric evaluation, the PADS was validated using two distinct adult samples: one comprising survivors of IPV, representing a clinical population at high risk for PAD, and another drawn from the general population to capture nonclinical variability in PAD. This approach allows for the examination of the scale's performance across different levels of symptom severity and relational contexts.

The validation process consisted of two studies, each focusing on specific psychometric properties: Study 1 evaluated the internal structure via exploratory and confirmatory factor analyses; Study 2 tested the factor structure via confirmatory factor analysis (CFA), assessed internal consistency and examined convergent and discriminant validity. Additionally, measurement invariance (MI) was explored to assess whether the scale operates equivalently across these populations, considering possible differences in item interpretation.

## 6 Study 1

Study 1 aimed to confirm and, if necessary, revise the theoretically proposed initial four‐factor structure of the state and trait versions of the PADS (Pugliese, Saliani, et al. 2023), including (1) the worry of losing one's dignity after separation from the abusive partner (named ‘Moral’), (2) the need to protect one's partner from suffering after separation (‘Altruistic’), (3) the fear of being alone and losing one's sense of safety after separation (‘Traumatic’) and (4) the internal conflict between separating because of one's suffering versus maintaining the harmful relationship at all costs (‘Internal Conflict’).

## 7 Method

### 7.1 Participants

A total of 360 participants (123 [34.2%] IPV survivors and 237 [65.8%] general population participants) took part in the study. We collected the following demographic variables: age, gender, sexual orientation, marital status, relationship characteristics, perceived economic dependence, treatment history and abuse type. Of the participants, 311 (87.0%) were females and 49 (13.0%) males; 215 (59.7%) were not married, 39 (10.8%) separated/divorced, 47 (13.0%) married and 59 (16.3%) widowed; a total of 262 (72.7%) were in a relation. Concerning their sexual orientation, 334 (92.8%) participants identified as heterosexual, 9 (2.5%) as homosexual, 11 (3.3%) as bisexual and 6 (1.4%) as having another sexual orientation. Participants' ages ranged from 19 to 73 years (M = 34.7; SD = 10.6). For those in a relationship, the duration of these relationships ranged from 2 months to 40 years (M = 7.4; SD = 6.9), with an average perceived economic dependence of 1.67 (SD = 1.1) on a Likert scale ranging between 1 (*not at all*) and 5 (*extremely*). In all, 65.3% of the sample had considered seeking professional help at least once, 80.3% were currently receiving care from a mental health professional and 90.0% of the partners were not under any form of psychological treatment.

For those participants who were not in a relationship, their most recent relationship ended on average 3.16 years (SD = 2.1) ago and had lasted on average 3.25 years (SD = 2.5). In total, 60.1% had considered seeking professional help at least once, 72.4% were under the care of a mental health professional during the past relationship and 78.5% of the ex‐partners were not. Of the 123 IPV survivors in the sample, 42 (34.1%) had suffered sexual abuse, 48 (39.0%) physical abuse and 123 (100%) psychological abuse.

### 7.2 Procedure

Demographic questions and the state and trait versions of the PADS were anonymously and voluntarily completed online using the Google Forms platform. While IPV nonsurvivors were recruited via a snowball procedure through social networks, IPV survivors were recruited with the collaboration of several anti‐violence organizations in Italy that shared the link to the study survey with IPV survivors. The study was conducted in accordance with the ethical standards of the Declaration of Helsinki, and completion of the survey took approximately 20 minutes. Informed consent was obtained from all participants (Ethical approval: Pr. 2/21).

### 7.3 Measures

The development of the PADS items was grounded in PAD's theoretical model (Pugliese, Saliani, et al. 2023), clinical interviews with individuals presenting with PAD and expert consultation with psychologists specializing in IPV and dependency. Initially, a pool of items was generated to capture key cognitive, emotional and relational aspects of PAD. Next, these items were refined through iterative expert feedback to ensure theoretical coherence, clarity and cultural appropriateness. This process resulted in a self‐report questionnaire comprising 39 items organized into four subscales named ‘Moral’ (Factor 1), ‘Altruistic’ (Factor 2), ‘Traumatic’ (Factor 3) and ‘Internal Conflict’ (Factor 4). Each subscale explores beliefs about the self, beliefs about the partner and core anti‐goals—i.e., the worst‐case scenarios that individuals with PAD try to avoid at all costs. Specifically, example items for the Moral subscale include ‘I feel invisible to my partner’ (self‐belief), ‘My partner devalues me’ (partner‐belief) and ‘I can't leave my partner because I am afraid I don't deserve someone better’ (anti‐goal). Example items for the Altruistic factor include self‐belief like ‘I am a very good person as I think of my partner's needs more than my own’, other belief such as ‘My partner is someone who constantly needs me’ and anti‐goal statements like ‘Separating from my partner is difficult because I can't bear the idea of his/her suffering’. Regarding the traumatic factor, examples of self‐belief include ‘I feel helpless in my relationship’, other beliefs such as ‘My partner mistreats me’ and anti‐goal like ‘Breaking up with my partner would make me feel lost’. For the internal conflict factor, examples include self‐belief such as ‘Others have noticed that since being with my partner, I frequently change my goals, plans, beliefs, but I am unaware of it’, other belief like ‘Sometimes, I feel satisfied in my relationship, but other times I weigh the possibility of completely breaking it off’ and anti‐goal such as ‘I am aware of the fact that my partner might not be the right person for me, but at the same time, I can't leave him/her’. The scale was administered two times with instructions that were adapted to measure state and trait PAD. Items are rated on a 5‐point Likert scale (1 = *not at all*, 2 = *slightly*, 3 = *sometimes*, 4 = *often*, 5 = *always*). The total score ranges between 39 and 195, with higher scores indicating greater PAD.

### 7.4 Statistical Analysis

A CFA was conducted to test the hypothesized four‐factor structure. A SEM analysis and the χ^2^/DF ratio (Wheaton et al. 1977), CFI, TLI (Bentler 1990; Hu and Bentler 1999) and RMSEA (Steiger 1990) as fit indices were employed. The following values were used as cut‐offs to determine the fit of the model: a value of χ^2^/DF lower than 2, an RMSEA lower than 0.05 and a minimum value of 0.95 for CFI and TLI (Hu and Bentler 1999; Bentler and Bonett 1980; Loehlin 2004). If the CFA rejected the hypothesized model, an exploratory factor analysis (EFA) was planned to derive an alternative factor structure. Kaiser–Meyer–Olkin (KMO) and Bartlett's indices were used to confirm sample adequacy and the correlation matrix factorability, followed by principal axis factoring (PAF). A screeplot analysis was used to determine the number of factors to be extracted. This method is considered reliable when the sample size is greater than 200 (Yong and Pearce 2013). The criterion that an eigenvalue higher than one should be used to determine the number of factors was discarded, since it can produce redundant factors (Velicer 1976). Since correlating factors were presumed, the rotation was done using the direct oblimin method (Kline 2014; Costello and Osborne 2005). The minimum saturation in the rotated solution was set at 0.32 to control for any cross‐loading (Tabachnick and Fidell 2007). The Jamovi software was used to perform the analysis (version 2.6.19). Results with a value of *p* < 0.05 are considered significant.

## 8 Results

**FIGURE 1:**
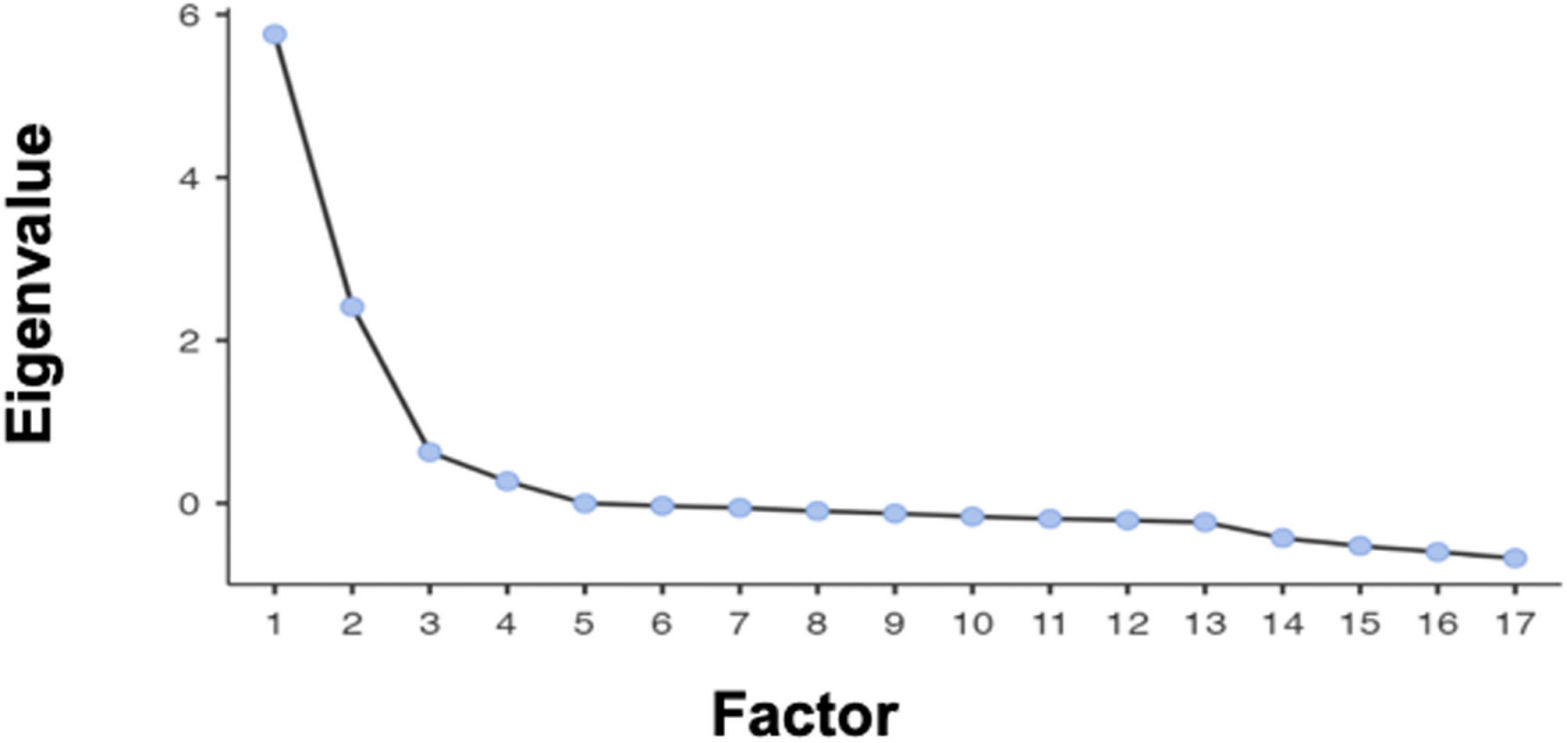
Screeplot generated from the exploratory factor analysis of the PADS state version (17 items).

**FIGURE 2:**
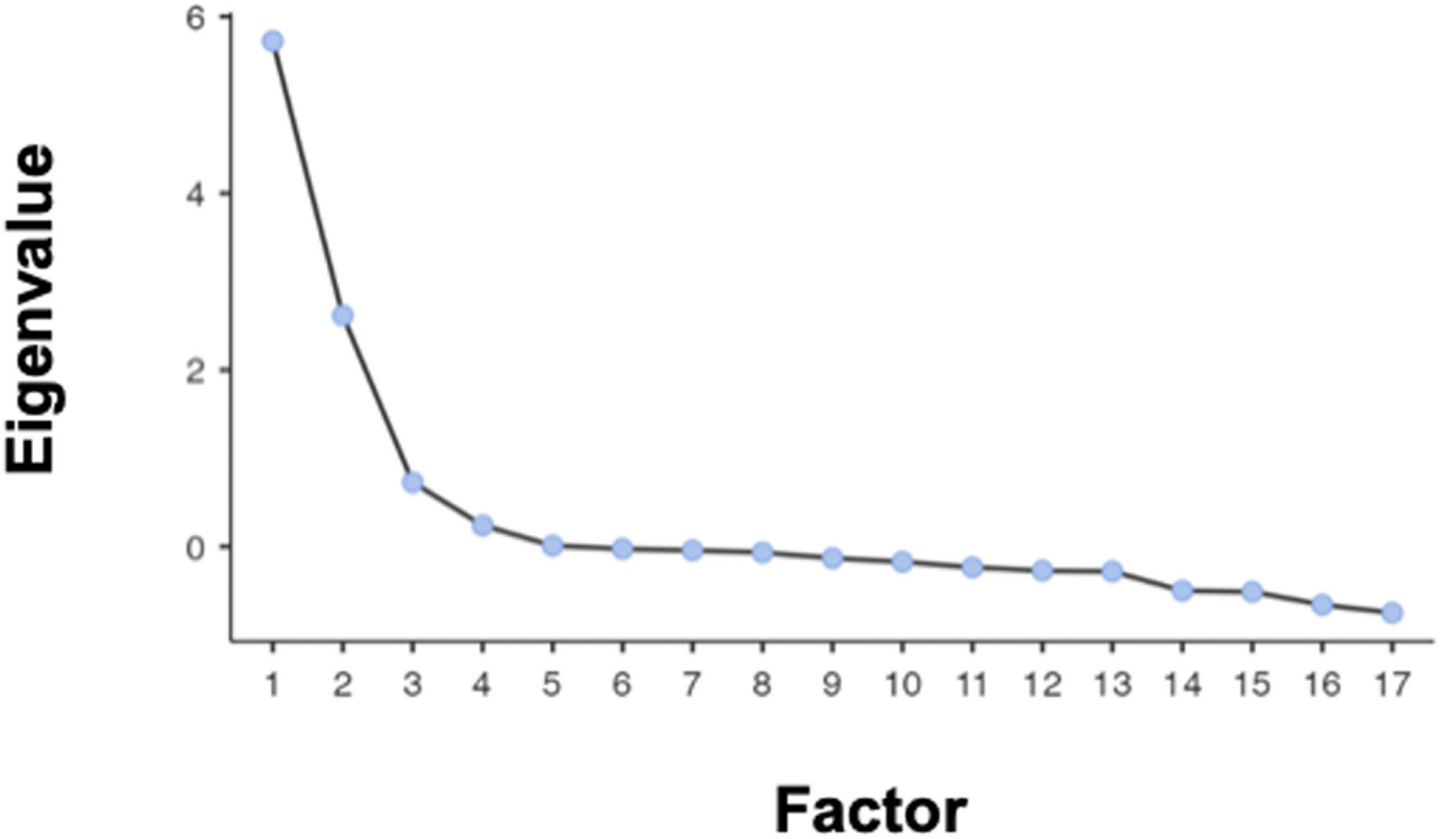
Screeplot generated from the exploratory factor analysis of the PADS trait version (17 items).

The CFA did not support the hypothesized four‐factor structure as the fit indices were not meeting the required cut‐offs: χ^2^/DF = 5.05, RMSEA = 0.106, CFI = 0.631 and TLI = 0.607 for the state version, and χ^2^/DF = 4.60, RMSEA = 0.100, CFI = 0.660 and TLI = 0.638 for the trait version. An EFA analysis was therefore conducted, which revealed a KMO of 0.910 for the state version and 0.899 for the trait version. The hypothesis of sphericity of the correlation matrix was rejected (*p* < 0.001). The screeplot suggested the presence of three factors in both the state and trait versions of the scale (Figures 1 and 2). To improve the scales, items with substantial cross‐loadings (above 0.30) or weak primary loadings (below 0.32) were removed. This resulted in a new version of the scales with 17 items that exhibited a factor loading of at least 0.35 and did not load on more than one factor in both the state and trait versions of the scale (Tables 1 and 2). The first factor, named ‘Internal Conflict’ (6 items), reflects the ambivalence about the quality of the relationship with the abusive partner and the ambivalence about the decision to separate or stay together (e.g., ‘Sometimes, I feel satisfied in my relationship, but at other times I consider the possibility of completely breaking it off’). The second factor, named ‘Inability to Separate’ (6 items), reflects the behavioural and psychological resistance to separation (e.g., ‘Leaving my partner would break me apart’). The third factor, named ‘Partner Abuse’ (5 items), reflects the abusive conditions in the relationship (e.g., ‘My partner mistreats me’).

**TABLE 1:** Factor loadings of the PADS trait version.

		Factor loading	
PADS item	**Factor 1**	**Factor 2**	**Factor 3**
37. I am aware of the fact that my partner might not be the right person for me, but at the same time, I cannot leave them	0.815		
38. I am aware that I deserve more love than what I receive, but at the same time, I cannot give up on my partner	0.803		
39. I am aware that I am suffering in this relationship, but at the same time, I cannot leave it	0.788		
34. Sometimes, I feel satisfied in my relationship, but other times I consider the possibility of completely breaking it off	0.755		
35. I find myself suddenly changing my mind about whether or not I want to be with my partner, based on their behaviour	0.657		
36. Sometimes I think I have the best person by my side, and then I change my mind and think the opposite	0.647		
28. Leaving my partner would break me apart		0.794	
27. Breaking up with my partner would make me feel lost		0.763	
30. I cannot live without my partner		0.731	
20. I would do anything to not lose the bond I have with my partner		0.668	
29. I could not leave my partner because despite everything they make me feel protected		0.641	
10. I would do anything in order to not lose my importance in my partner's eyes		0.579	
4. My partner devalues me			0.828
26. My partner mistreats me			0.814
24. My partner manipulates me			0.711
6. My partner behaves as if I were invisible			0.650
5. My partner makes me feel as if I am mistaken			0.635

**TABLE 2:** Factor loadings of the PADS state version.

		Factor loading	
PADS item	**Factor 1**	**Factor 2**	**Factor 3**
39. I am aware that I am suffering in this relationship, but at the same time, I cannot leave it	0.811		
38. I am aware that I deserve more love than what I receive, but at the same time, I cannot give up on my partner	0.788		
37. I am aware of the fact that my partner might not be the right person for me, but at the same time, I cannot leave them	0.785		
34. Sometimes, I feel satisfied in my relationship, but other times I weigh the possibility of completely breaking it off	0.776		
35. I find myself suddenly changing my mind about whether or not I want to be with my partner, based on their behaviour	0.767		
36. Sometimes I think I have the best person by my side, and then I change my mind and think the opposite	0.673		
27. Breaking up with my partner would make me feel lost		0.817	
28. Leaving my partner would break me apart		0.811	
20. I would do anything to not lose the bond I have with my partner		0.716	
30. I cannot live without my partner		0.695	
29. I could not leave my partner because despite everything they make me feel protected		0.688	
10. I would do anything in order to not lose my importance in my partner's eyes		0.605	
26. My partner mistreats me			0.786
5. My partner makes me feel as if I am mistaken			0.782
24. My partner manipulates me			0.695
4. My partner devalues me			0.675
6. My partner behaves as if I were invisible			0.550

## 9 Study 2

The purpose of Study 2 was twofold: first, to validate the factor structure and assess the internal consistency of the 17‐item PADS, developed in Study 1, in an independent sample of clinical (IPV survivors) and nonclinical (non‐IPV survivors) individuals through CFA; second, to analyse the convergent and discriminant validity of both the state (PADS‐S) and trait (PADS‐T) versions of the 17‐item PADS by examining their associations with established measures of depression, relationship quality and intimate partner characteristics, and to explore MI.

In line with the cognitive model of PAD (Pugliese, Saliani, et al. 2023), we tested the following hypotheses:

- Positive correlation between the PADS (PADS‐T and PADS‐S) and Beck Depression Inventory‐II (BDI‐II) as a measure of depression (see below). This hypothesis is based on findings from a pilot study on PADS and the cognitive model of PAD (Pugliese, Saliani, et al. 2023; Pugliese, Mosca, et al. 2023), as well as recent research on the positive relationship between IPV and depression (Bacchus et al. 2018; Chandan et al. 2019).
- Negative correlation between the PADS (PADS‐T and PADS‐S) and Perceived Relationship Quality Components (PRQC) as a measure of perceived relationship satisfaction (see below). This hypothesis is based on the suffering and difficulty associated with being in or leaving abusive relationships for individuals with PAD.
- Stronger correlations between PADS‐S and Trait‐Specific Dependence Inventory (TSDI) scores, reflecting the influence of current partner traits on situational manifestations of PAD. In contrast, PADS‐T was expected to show weaker or nonsignificant associations, as it captures a more stable predisposition that is less influenced by present relational dynamics (Pugliese, Mosca, et al. 2023).

## 10 Method

### 10.1 Participants

A new sample of 362 participants (121 [33.0%] survivors of violence, 241 [67.0%] general population participants) with an age range of 18–79 years (M = 36.0; SD = 13.3) (230 [64.0%] females, 132 [36.0%] males; 53.3% not married; 8.3% separated/divorced; 27.6% married; 10.8% widowed; 78.0% in a relation; 35.0% cohabiting partners; 91.0% heterosexuals; 2.5% homosexuals, 4.9% bisexuals; and 1.4% other) was used. We collected the following demographic variables: age, gender, sexual orientation, marital status, relationship characteristics, perceived economic dependence, treatment history and abuse type. For those in a relationship, the duration ranges between 3 months and 40 years (M = 9.4; SD = 11.1), with a perceived average economic dependence of 1.6 (SD = 1.1; ranging between 1 and 5). In 70.5% of the sample had considered seeking professional help at least once; 78.9% were under the care of a mental health professional, and 91.2% of the partners were not. For those who were not in a relationship, the last relationship ended by 2.44 years (SD = 2.1) and lasted 3.92 years (SD = 2.4). In total, 59.3% had considered seeking professional help at least once, 70.4% were under the care of a mental health professional during the past relationship and 76.5% of the ex‐partners were not. Of the 121 survivors, 32.2% have suffered sexual abuse, 33.8% physical abuse and 100% psychological abuse.

### 10.2 Procedures and Measures

The procedures were identical to those in Study 1 (see above), except that the 17‐item version of the PADS was administered. In addition to the PADS, all participants completed the following measures:

Beck Depression Inventory‐Second Edition (BDI‐II; Ghisi et al. 2006): The BDI‐II is a 21‐item self‐report questionnaire used to measure the severity of depression. Items are rated on a 4‐point Likert scale (0 = *not at all*, 3 is different for each item). Total scores range from 0 to 63, with scores of 0 to 13 indicating minimal depression, 14 to 19 indicating mild depression, 20 to 28 indicating moderate depression and 29 to 63 indicating severe depression. Cronbach's alpha was 0.81 in the original study (Beck et al. 1996) and 0.94 in the present study.

Perceived Relationship Quality Components Scale (PRQC; Fletcher et al. 2000): The PRQC consists of 18 questions that assess six relationship dimensions, including satisfaction, commitment, intimacy, trust, passion and love. Each dimension is evaluated using three questions rated on a 7‐point Likert scale (1 = *not at all*, 7 = *definitely*). The total score ranges from 18 to 126, with higher scores indicating better relationship quality. In the original study, the α was 0.88; in our study, the α was 0.95.

Trait‐Specific Dependence Inventory (TSDI; Ellis et al. 2002): The TSDI is a 35‐item self‐report questionnaire that assesses six intimate partner characteristics, including agreeable/committed, resource accruing potential, physical prowess, emotional stability, surgency and physical attractiveness. Items are rated on a 5‐point Likert scale (1 = *not difficult at all*, 5 = *extremely difficult*). The total score ranges from 35 to 175, with higher scores indicating more positive characteristics of the partner. In the original study, the α ranged from 0.75 to 0.91; in our study, it was 0.98.

### 10.3 Statistical Analysis

The consistency of the three‐factor model was assessed with the same fit indexes as in Study 1. The following values were used as cut‐offs to determine the fit of the model: a value of χ^2^/DF lower than 2, an RMSEA lower than 0.05 and a minimum value of 0.95 for CFI and TLI (Hu and Bentler 1999; Bentler and Bonett 1980; Loehlin 2004). Modification Indexes were used to optimize the model. Residual covariances were added, when necessary, between items loading on the same factor (Bollen 1989; Hu and Bentler 1999). These modifications were theoretically justified by assuming common sources of shared variance, such as semantic similarity or content overlap between items. According to Stevens (1996), the sample size used for the SEM analysis requires at least 15 observations for each measured variable, while other authors (Bentler and Chou 1987) suggest a minimum of five cases for each parameter estimated by the model. Our sample size met both criteria. The reliability analysis of the scales emerging from the CFA was assessed with Cronbach's alpha (Cronbach 1951), using a reference interval with values between 0.70 and 0.95, considering that values beyond the upper boundary are indicative of redundant items (Bland and Altman 1997; Tavakol and Dennick 2011). Convergent and discriminant validity of the PADS were determined by computing Spearman correlations of the PADS with the other measures, and MI was determined by multigroup analysis. All analyses were performed with the Jamovi software (version 2.6.19). Results with a value of *p* < 0.05 are considered significant.

## 11 Results

**FIGURE 3:**
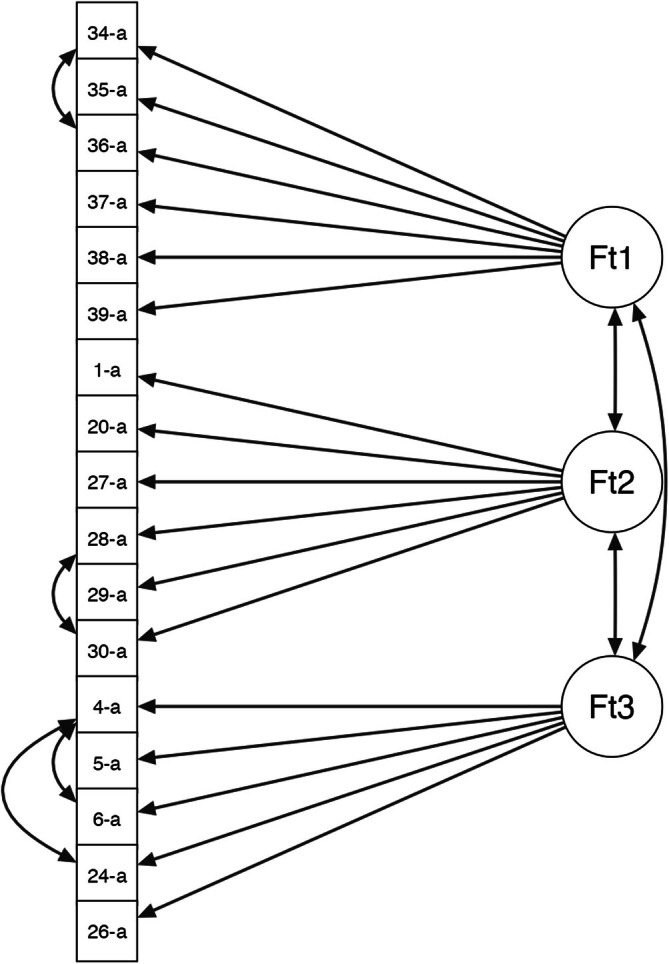
Path diagram of the optimized model of the PADS state version.

**FIGURE 4:**
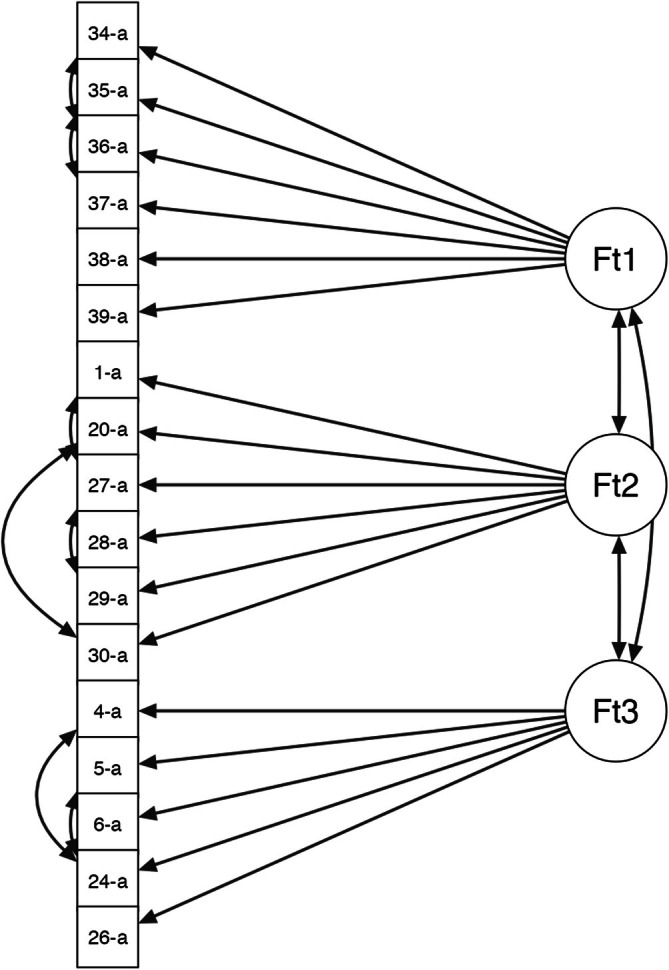
Path diagram of the optimized model of the PADS trait version.

The hypothesized three‐factor structure of the state and trait versions of the scale met the cut‐offs on none of the fit indices; therefore, it was improved by introducing covariances between some items within the same factor (Figures 3 and 4). In the first factor (‘Internal Conflict’) of both versions, we introduced, for example, a covariance between ‘I find myself suddenly changing my mind about whether or not I want to be with my partner, based on his/her behavior’ and ‘Sometimes I think I have the best person by my side, and then I change my mind and think the opposite’; in the second factor (‘Inability to Separate’), there was a covariance between ‘I would do anything in order to not lose my importance in my partner's eyes’ and ‘I would do anything to not lose the bond I have with my partner’; and in the third factor (‘Partner Abuse’), there was a covariance between ‘My partner devalues me’ and ‘My partner manipulates me’. After these modifications, the three‐factor structure of the 17 items was confirmed for the state and trait versions of the scale, as all the covariances were significant and the modification indices did not suggest further improvements. As shown in Tables 3 and 4, the value of the χ^2^ statistic decreased compared to the original model but remained nonsignificant. Overall, the goodness of fit was satisfactory: The χ^2^/DF ratio was well below the value of 2, and the RMSEA was below the threshold of 0.05. Finally, the CFI and TLI indices had values above 0.95.

**TABLE 3:** Absolute and comparative fit indices of the original and optimized PADS state version.

	Original model	Optimized model
Absolute fit indices		
χ^2^ (df.; sign.)	567 (134; *p* < 0.001)	117 (106; *p* < 0.001)
χ^2^/DF	4.23	1.10
RMSEA	0.0945	0.0167
Comparative fit indexes		
CFI	0.845	0.997
TLI	0.823	0.996

**TABLE 4:** Absolute and comparative fit indexes of the original and optimized PADS trait version.

	Original model	Optimized model
Absolute fit indexes		
χ^2^ (df.; sign)	557 (134; *p* < 0.001)	139 (101; *p* < 0.001)
χ^2^/DF	4.15	1.37
RMSEA	0.0934	0.0323
Comparative fit indexes		
CFI	0.831	0.989
TLI	0.807	0.985

The PADS total and subscales showed satisfactory internal consistency (Tables 5 and 6). No items, if excluded, would have increased Cronbach's alpha.

**TABLE 5:** Cronbach's alpha for the total scale and the distinct factors of the PADS state version.

	No. of items	Cronbach's alpha
PADS state	17	0.891
Factor 1: internal conflict	6	0.904
Factor 2: inability to separate from the abusive partner	6	0.851
Factor 3: partner abuse	5	0.914

**TABLE 6:** Cronbach's alpha for the whole trait version scale and the distinct factors.

	No. of items	Cronbach's alpha
PADS trait	17	0.890
Factor 1: internal conflict	6	0.884
Factor 2: inability to separate from the abusive partner	6	0.843
Factor 3: partner abuse	5	0.894

The average total score of the PADS‐Trait was 33.00 (SD = 12.45), of the PADS‐State 34.4 (SD = 13.67) and of the BDI‐II 14.16 (SD: 12.32). Convergent validity was confirmed by significant Spearman's *ρ* values (BDI‐II and PADS‐T: 0.401, *p* < 0.001; BDI‐II and PADS‐S: 0.421, *p* < 0.001). The average total score of the PRQC was 92.5 (SD: 25.4). The convergent validity was confirmed by Spearman's *ρ* (PRQC and PADS‐T: −0.377, *p* < 0.001; PRQC and PADS‐S: −0.520, *p* < 0.001; Table 7). The average total score of the TSDI was 55.39 (SD: 27.16). The discriminant validity was confirmed by Spearman's *ρ* (TDSI and PADS‐T: 0.06, *p* = 0.25), and a positive correlation was found between TSDI and PADS‐S (0.25, *p* < 0.001; Table 7).

**TABLE 7:** Convergent validity of the PADS state and trait versions.

	PADS state	PADS trait
BDI‐II	0.421***	0.401***
PRQC	−0.520***	−0.377***
TSDI	0.25***	0.06

Abbreviation: BDI‐II = Beck Depression Inventory‐II.

*** *p* < 0.001.

Finally, we explored MI of the PADS across IPV survivors and nonsurvivors using chi‐square difference testing. The fit indices did not support invariance across groups at the tested levels, suggesting potential differences in how the construct is perceived or expressed between survivors and nonsurvivors. Full statistical details are provided in Tables S8–S10 for transparency and due to space limitations in the main text.

## 12 Discussion

The present study aimed to develop and validate a new instrument that assesses both state and trait PAD, a psychological construct characterized by an internal conflict between the desire to stay in a harmful relationship and the need to separate for safety. While the state captures situational and context‐dependent expressions of affective dependence, the trait reflects a more stable and enduring predisposition toward such relational dynamics. The resulting instrument, the PADS, consists of 17 items and was intentionally designed to balance psychometric robustness with clinical usability. This consideration is especially important when working with IPV survivors and individuals exhibiting PAD, who frequently report chronic stress, emotional dysregulation and trauma‐related symptoms (Amell et al. 2022; Pugliese et al. 2024; Silvestri et al. 2025), all of which can impair their capacity to engage with lengthy or cognitively demanding assessments.

The factor analytic findings revealed a structure that effectively captures the core phenomenology of PAD. Specifically, this structure includes three distinct factors: ‘Internal Conflict’ (Factor 1), ‘Inability to Separate’ (Factor 2) and ‘Partner Abuse’ (Factor 3) in both the state and trait versions of the PADS. The items in the ‘Internal Conflict’ factor reveal a profound struggle within individuals with PAD. Items such as ‘I am aware of the fact that my partner might not be the right person for me, but at the same time, I can't leave him/her’ and ‘I find myself suddenly changing my mind about whether or not I want to be with my partner, based on his/her behavior’ underscore the psychological tension individuals face when attempting to reconcile the awareness of an unhealthy relationship with the difficulty of disengagement (Dutton and Painter 1993), in line with what the theory of PAD (Pugliese, Saliani, et al. 2023) postulated. In the ‘Internal Conflict’ factor, only items describing alternate and akratic conflict reached the psychometric requirements for inclusion. The exclusion of absent conflict, involving a lack of awareness, stems from its poor measurability via self‐report instruments. This oscillation between denial and awareness is a hallmark of PAD and aligns with trauma‐related psychological processes (Dutton and Painter 1993; Hulley et al. 2023). The ‘Inability to Separate’ factor delves into the specific challenges individuals encounter when contemplating leaving an abusive relationship. The term ‘inability’ is used not to imply a literal lack of agency, but rather to highlight the perceived emotional incapacitation to separate, a core characteristic of the condition of IPV survivors (Anderson and Saunders 2003). The items within this factor, such as ‘Breaking up with my partner would make me feel lost’ and ‘Leaving my partner would break me apart’, emphasize the profound emotional and psychological impact associated with the idea of separation. The third factor, ‘Partner Abuse’, explores the impact of the abusive partner's behaviour on the victim's emotional state. Items like ‘My partner devalues me’ and ‘My partner makes me feel as if I am mistaken’ reflect the detrimental aspects of the partner's actions. This factor shows the centrality of the partner's abusive, mistreating and manipulative behaviours in shaping the overall experience of PAD (Pico‐Alfonso et al. 2006).

Regarding convergent validity, as expected, depression was connected to both state and trait PAD. These findings align with our clinical observations and a previous pilot study that used the PADS (Pugliese, Mosca, et al. 2023). They also match results from several earlier studies highlighting the link between depression and the physical and mental well‐being of IPV survivors (Pico‐Alfonso et al. 2006; Bacchus et al. 2018; Oram et al. 2022). Another recent study (Chandan et al. 2019) also found a relationship between PAD and depression, showing that IPV survivors experienced higher levels of both depression and anxiety compared to nonsurvivors. It is important to note that since correlation does not imply causation, more research is needed to understand the connection between PAD and depression. The negative correlation between PAD (state and trait) and relationship satisfaction further supports the PADS' convergent validity, as individuals with PAD often suffer yet remain in abusive relationships (Hammett et al. 2021). Discriminant validity was assessed by examining the correlation between the PADS and partner characteristics, measured using the TSDI (Ellis et al. 2002). The results show no correlation between the PAD trait and partner characteristics, while the PAD state exhibits a positive correlation. Like other constructs that exist as both stable traits and situational states (McCrae and Costa 2008), the PAD trait seems relatively independent of contextual factors, whereas the PAD state appears more sensitive to current life circumstances, including characteristics of an intimate partner (Pugliese, Mosca, et al. 2023).

The lack of invariance between the samples suggests that survivors and nonsurvivors might interpret the PADS items differently. This result may reflect not a psychometric flaw, but genuine differences in trauma‐related meaning‐making across populations (Welzel et al. 2023). Sample‐specific characteristics, such as prior exposure to abuse, levels of dissociation, shifts in how individuals perceive or report symptoms based on contextual and emotional variables or varying degrees of insight into the abusive dynamic, may have contributed to the observed discrepancies (Lommen et al. 2014). This suggests that measurement properties can vary across groups, not because of flaws in the instrument, but due to genuine psychological differences in how trauma is processed and disclosed.

Importantly, the PADS has demonstrated satisfactory psychometric properties in both victim and general population samples, supporting its broader validity. The lack of full invariance does not invalidate the scale's clinical or theoretical utility but rather underscores the need for continued research to refine its applicability across diverse subgroups. Overall, the PADS represents a theoretically grounded and clinically valuable instrument for assessing a critical—yet often overlooked—dimension of relational trauma in survivors of IPV.

### 12.1 Clinical and Research Implications

The PADS represents a promising tool for the early identification of key mechanisms underlying PAD—namely, internal conflict, inability to separate and partner abuse. Clinically, these findings underscore the importance of addressing both trait PAD, conceptualized as a chronic predisposition to dysfunctional relational patterns, and state PAD, understood as context‐dependent intensifications of affective dependence triggered by specific partner dynamics. This distinction facilitates more nuanced assessments and individually tailored interventions across therapeutic settings.

Insights into the origins of PAD—particularly its roots in early relational trauma—are especially valuable in psychoeducational contexts, where survivors often struggle to understand the psychological processes behind their entrapment. Many engage in self‐blame for not leaving earlier, even when they are cognitively aware of clear warning signs (Anderson and Umberson 2001). Clarifying the cognitive‐emotional structure of PAD can help clinicians guide survivors toward a more compassionate and integrated understanding of their experience, potentially improving therapeutic engagement and emotional recovery.

In addition, the PADS may prove useful in risk assessment protocols. Although clinical cut‐off scores have not yet been established, elevated responses—particularly on the internal conflict and inability to separate subscales—may indicate heightened vulnerability to remaining in or returning to an abusive relationship, a well‐documented risk factor for recurrent IPV and femicide (Monckton Smith 2020). Consequently, the scale could be integrated into multidisciplinary frameworks involving mental health services, social work and law enforcement.

From a research perspective, the PADS opens new avenues for investigating the psychological mechanisms that sustain affective dependence in violent relationships. It also allows for the identification of distinct profiles of IPV survivors with PAD, based on which basic psychological needs—such as love, dignity or safety—are most frustrated. Furthermore, the scale provides a tool for exploring the role of PAD not only in victimization but also in IPV perpetration, in which, perhaps counter‐intuitively, PAD may also play a role. These future research directions may inform both the development of targeted interventions and the refinement of theoretical models of relational violence.

### 12.2 Limitations and Future Directions

This study has several limitations. First, trauma‐related symptoms in the IPV sample—such as hypervigilance and emotional numbing (Näätänen et al. 2002)—may have affected the accuracy of self‐reported PAD. Furthermore, the absence of measures assessing trauma‐related symptoms, including dissociation, may confound associations between PAD and IPV, as emotional numbing may reduce self‐reported dependence, while dissociative experiences might distort attachment behaviours. Without accounting for these variables, it remains unclear whether PAD reflects a pre‐existing trait or a consequence of IPV or unresolved trauma. Future studies should include validated measures of post‐traumatic stress disorder, dissociation and other potential confounding variables to clarify these relationships and strengthen theoretical and clinical conclusions.

Second, the overrepresentation of women limits the generalizability of our findings to other gender identities, as gender norms may influence how PAD is experienced and reported (Bates et al. 2019). Future research should include more gender‐balanced samples to explore potential differences.

Third, certain psychometric properties of the PADS also require further investigation. Specifically, test–retest reliability has not yet been assessed; normative data and clinical cut‐off scores are still needed to enhance practical utility.

Fourth, while dyadic assessments can clarify objective partner behaviours, it is important to note that the Partner Abuse subscale captures subjective perceptions rather than externally validated abuse. This aligns with the theoretical conceptualization of PAD as a psychological condition characterized by perceiving the partner as harmful or rejecting, which contributes to maladaptive dependence and relational entrapment. Furthermore, our study population partly included IPV survivors who were recruited through anti‐violence centres, where partner abuse is professionally assessed.

Fifth, a further limitation concerns the absence of detailed information regarding participants' psychotherapeutic history. Although we collected data on whether the individuals and/or their partners had previously engaged in psychotherapy, we did not systematically assess key aspects such as the therapeutic orientation, duration or reasons for treatment. This lack of specificity prevented us from accounting for the potential influence of past psychotherapeutic experiences, such as trauma‐focused CBT, EMDR or supportive therapy, which may significantly affect symptomatology and psychological functioning, including manifestations of PAD.

Sixth, although the broad age range of participants may enhance the generalizability of our findings, it is possible that specific age‐related factors—such as somatic health problems, cognitive decline or low literacy levels—could have influenced participants' responses. As these variables were not assessed or controlled for in the present study, future research should consider examining their potential impact.

Additional research should address the cross‐cultural validity of the scale and examine longitudinal trajectories of PAD in both survivors and perpetrators of IPV. Investigating the responsiveness of PAD to treatment and the role of partner characteristics in shaping state PAD may inform the development of more effective therapeutic protocols grounded in the PAD model.

## Author Contributions

Conceptualization: P.E. Writing – original draft preparation: P.E. and U.A. Writing – review and editing: P.E., U.A., A.A. and E.A. Visualization: V.T., Q.C. and C.E. Supervision: A.A., E.A., S.A.M. and F.F. Project administration: P.E., F‐B.M.G., V.T. and M.F. All authors have read and agreed to the published version of the manuscript.

## Conflicts of Interest

The authors declare no conflicts of interest.

## Supporting information


**Table S8:** Measurement invariance results.
**Table S9:** Factorial covariances for the PADS trait version.
**Table S10:** Factorial covariances for the PADS state version.

## Data Availability

The data that support the findings of this study are available from the corresponding author upon reasonable request.
