# Supplementary material for: Pathological Affective Dependence as a Risk Factor for Intimate Partner Violence: Initial Psychometric Validation of the Italian Version of the Pathological Affective Dependence Scale
Source: Clin Psychol Psychother. 2025 Aug 15;32(4):e70140. doi: 10.1002/cpp.70140 (PMC12357130; doi:10.1002/cpp.70140)
Supplement: Supplementary file 1 — Table S8: Measurement invariance results. Table S9: Factorial covariances for the PADS trait version. Table S10: Factorial covariances for the PADS state version. [file CPP-32-e70140-s001.docx]

**Supplementary materials**

**Table 8**

*Measurement invariance results*

| **STATE** | | | | | |
| --- | --- | --- | --- | --- | --- |
| **Multigroup analysis - victims vs non-victims** | | | | | |
|  | **Chi square** | **DF** | **Chi square diff.** | **DF diff.** | **p value** |
| **Model 1 - unconstrained** | 378,75 | 224 |  |  |  |
| **Equal regression weights** | 430,192 | 238 | 51,442 | 14 | 0,000 |
| **Equal regression weights and covariances** | 606,165 | 244 | 227,415 | 20 | 0,000 |
| **Equal regression weights and covariances and residuals** | 733,222 | 265 | 354,472 | 41 | 0,000 |
| **TRAIT** | | | | | |
| **Multigroup analysis - victims vs non-victims** | | | | | |
|  | **Chi square** | **DF** | **Chi square diff.** | **DF diff.** | **p value** |
| **Model 1 - unconstrained** | 268,394 | 202 |  |  |  |
| **Equal regression weights** | 320,171 | 216 | 51,777 | 14 | 0,000 |
| **Equal regression weights and covariances** | 459,459 | 222 | 191,065 | 20 | 0,000 |
| **Equal regression weights and covariances and residuals** | 654,981 | 254 | 386,587 | 52 | 0,000 |

**Table 9**

*Factorial covariances for the PADS trait version*

|  |  | **Estimate** | **SE** | **Z** | **p** |
| --- | --- | --- | --- | --- | --- |
| **Factor 1** | Factor 1  Factor 2  Factor 3 | 1.000*  0.301  0.825 | 0.0546  0.0252 | 5.52  32.71 | <.001  <.001 |
| **Factor 2** | Factor 2  Factor 3 | 1.000*  0.214 | 0.0576 | 3.71 | <.001 |
| **Factor 3** | Factor 3 | 1.000* |  |  |  |

*= fixed parameter

**Table 10**

*Factorial covariances for the PADS state Version*

|  |  | **Estimate** | **SE** | **Z** | **p** |
| --- | --- | --- | --- | --- | --- |
| **Factor 1** | Factor 1  Factor 2  Factor 2 | 1.000*  0.184  0.825 | 0.0597  0.0222 | 3.08  37.21 | 0.002  <.001 |
| **Factor 2** | Factor 2  Factor 3 | 1.000*  0.113 | 0.0600 | 1.88 | 0.060 |
| **Factor 3** | Factor 3 | 1.000* |  |  |  |

*= fixed parameter
